# Supplementary material for: Effects of Mitochondrial Transplantation on Transcriptomics in a Polymicrobial Sepsis Model
Source: Int J Mol Sci. 2023 Oct 18;24(20):15326. doi: 10.3390/ijms242015326 (PMC10607172; doi:10.3390/ijms242015326)
Supplement: Supplementary file 1 [file ijms-24-15326-s001.zip › Transcriptomics_Mt TPL ver. 1.0_Supp_Figures.pdf]

Supplementary Materials

Effects of Mitochondrial Transplantation on Transcriptomics in a Polymicrobial Sepsis Model

Seongmin Kim [1,†], Ji Heon Noh [2,†], Min Ji Lee [3], Ye Jin Park [3], Bo Mi Kim [3], Yun-Seok Kim [3], Sangik Hwang [2], Chungoo Park [1,*] and Kyuseok Kim [3,*]

[1] School of Biological Science and Technology, Chonnam National University, Gwangju 61186, Republic of Korea; sgmn0223@gmail.com
[2] Department of Biochemistry, Chungnam National University, Daejeon 34134, Republic of Korea; journi@cnu.ac.kr (J.H.N.), hsi9740@naver.com (S.H.)
[3] Department of Emergency Medicine, CHA University School of Medicine, Seongnam 13497, Republic of Korea; minji.lee29@gmail.com (M.J.L.); yejin6577@naver.com (Y.J.P.); alfks4050@naver.com (B.M.K.); loupys@naver.com (Y.-S.K.)
* Correspondence: chungoo@jnu.ac.kr (C.P.); dreinstein70@gmail.com or dremkks@cha.ac.kr (K.K.)
† These authors equally contributed to this work.

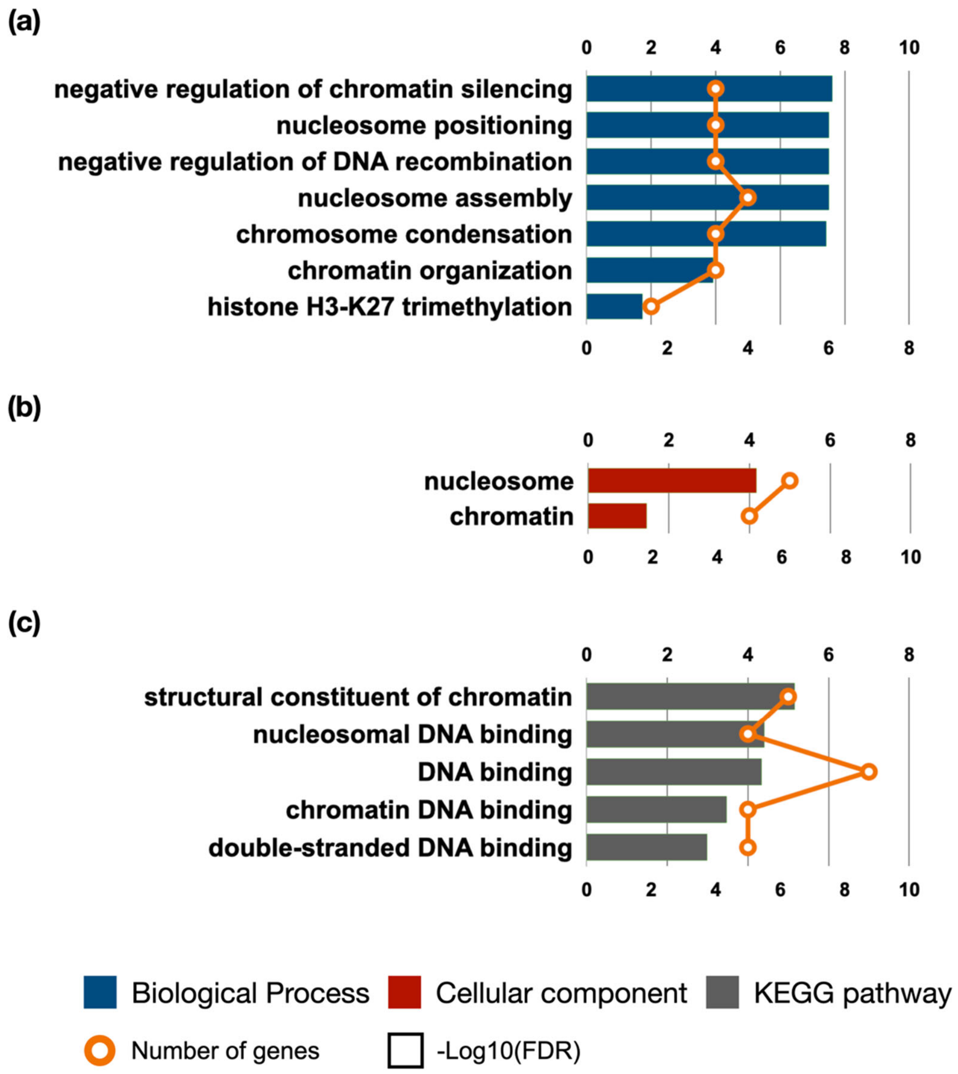

Figure S1. The significantly enriched Gene ontology (GO) biological process (**a**) and cellular component (**b**) categories and KEGG (**c**) pathways of 160 DEGs that were significantly upregulated in SEPSIS versus SEPSIS + MT.

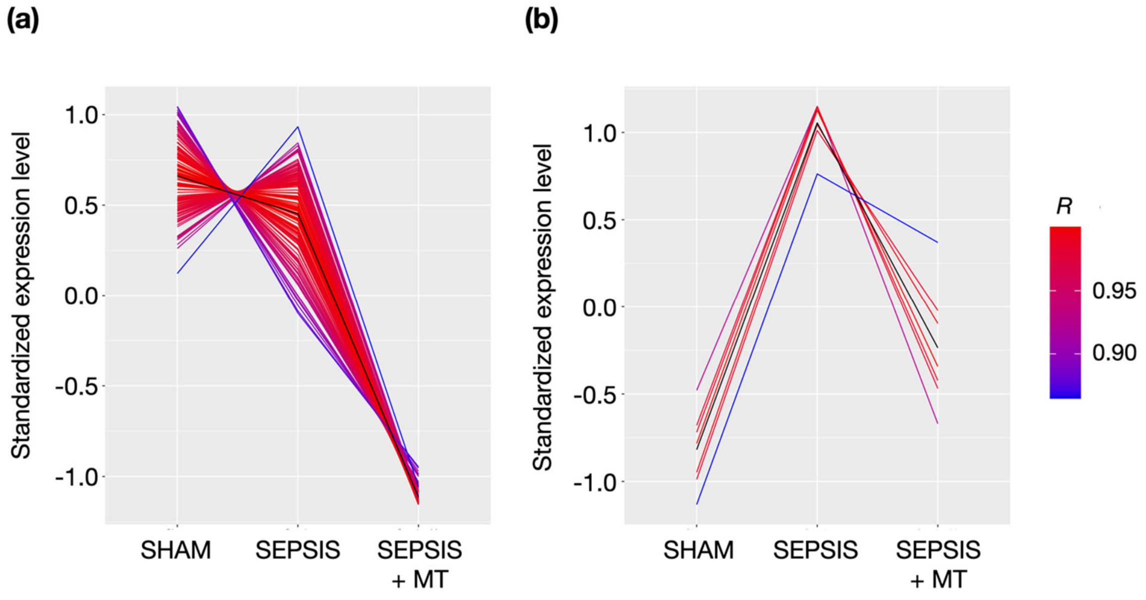

Figure S2. Trajectories of 160 DEGs expression change in three different groups. 153 genes were represented in cluster 1 (**a**) and the others (seven genes) were identified in cluster 2 (**b**). Black and other colored lines represent the centroid of clustered gene expression levels and each their own one, respectively. The gradient scale shows the value of Pearson's correlation coefficients (*R*).

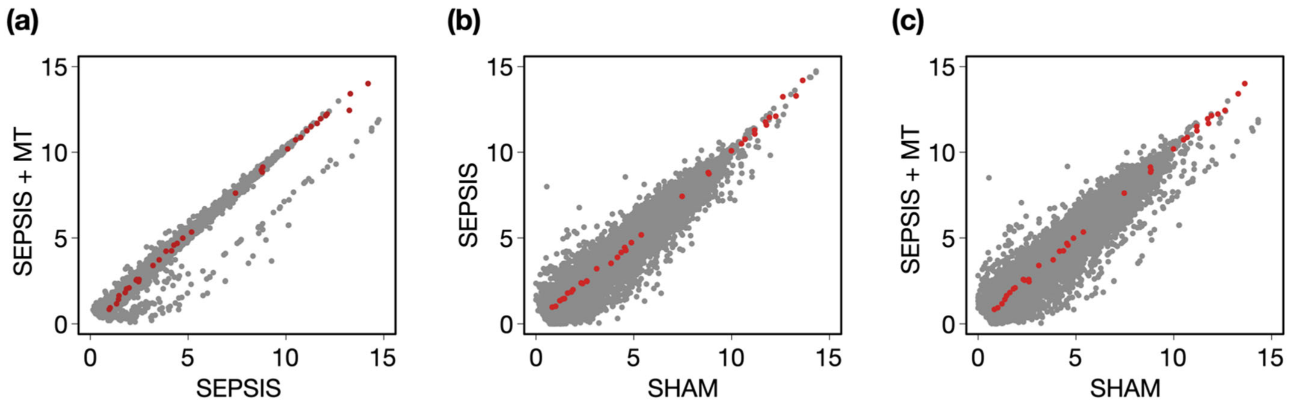

Figure S3. Scatter plots of RNA-seq data between SEPSIS and SEPSIS + MT (**a**), between SEPSIS and SHAM (**b**), and between SEPSIS + MT and SHAM (**c**). Red dot indicates the gene of mitochondrial genome.
